# Effectiveness of a manual dexterity training program to improve executive functioning in preschool children: an individual difference analysis

**DOI:** 10.3389/fcogn.2025.1433759

**Published:** 2025-03-28

**Authors:** Christina Stuhr, Charmayne Mary Lee Hughes, Tino Stöckel

**Affiliations:** ^1^Sport and Exercise Psychology Unit, Department of Sport Science, University of Rostock, Rostock, Germany; ^2^Age-Appropriate Human-Machine Systems, Institute of Psychology and Ergonomics, Technische Universität Berlin, Berlin, Germany

**Keywords:** executive functions, child development, reliable change index, manual dexterity, preschool

## Abstract

**Introduction:**

The present study employed the Jacobson-Truax reliable change index (RCI) to examine the effectiveness of a 4-week manual dexterity training program embedded in a socially enriched group setting to improve working memory performance, cognitive functioning, and numeracy skills in preschool children.

**Methods:**

Forty-five typically developing children aged between 5 and 6 years of age were randomly allocated to a 4-week intervention program (*n* = 20) or a control condition (*n* = 25). Pre- and post-test assessments were conducted using two manual dexterity measures, three working memory measures, as well as tasks evaluating inhibition, cognitive flexibility, processing speed, and numeracy skills.

**Results:**

Relative to control participants, a greater number of participants in the intervention group showed statistically and clinically significant post-intervention gains in manual dexterity, working memory, and selective attention. However, the benefits of the intervention did not extend to response inhibition, cognitive flexibility, processing speed, and numeracy skills. Moreover, a greater percentage of children in the intervention group exhibited improvements in both manual dexterity and all working memory tasks than children in the control group.

**Discussion:**

Overall, findings from this study corroborate existing research demonstrating the positive effects of manual dexterity training on working memory performance and highlight the use of individual-level analysis to determine the effectiveness of cognitive-motor training and interventions.

## 1 Introduction

Executive Functions (EFs) are multiple, interrelated cognitive abilities (Anderson, 2008; Diamond, 2013) that are used when thoughts and movements must be consciously controlled to act in a goal-oriented manner. EFs are especially relevant in situations requiring concentration and thinking, when remaining on “autopilot,” or relying on instincts would not lead to success (Burgess and Simons, 2005; Diamond, 2013; Miller and Cohen, 2001). Diamond (2013) defined three core EFs: working memory (temporary storage and manipulation of information in mind), inhibitory control (the regulation of attention, motivation, thoughts, and behavior), and cognitive flexibility (the ability to change perspectives). Empirical research has indicated that EF performance is related to mathematics (Fuhs et al., 2014; Hernández et al., 2018) and reading outcomes (Cirino et al., 2019; Lenes et al., 2020), and is a better predictor of later school success than the intelligence quotient (IQ, Alloway and Alloway, 2010; Cameron et al., 2015; Michel et al., 2019).

Children with low working memory exhibit deficits in knowledge and skill acquisition (Swanson and Alloway, 2012), often fail classroom activities (Gathercole and Alloway, 2008), and fail to catch up academically with their peers, even after controlling for IQ (Alloway and Alloway, 2010). It is likely for this reason that there has been a wealth of research evaluating the efficacy of school- or computer-based working memory interventions (Alloway, 2012; Colmar et al., 2016; Loosli et al., 2012). For example, Loosli et al. (2012) evaluated the ability of a 2-week adaptive working memory-based training intervention to improve working memory and reading performance in children aged 9–11 years. In comparison to the control group, children that received the intervention significantly improved their performance in the trained working memory task, as well as their single word and text reading performance.

Other researchers have focused on developing motor training interventions to improve working memory and EF performance during childhood (Lin et al., 2021), pointing to neurophysiological evidence indicating that motor and cognitive processes are controlled by the same or overlapping brain areas [e.g., pre-frontal cortex (PFC) and cerebellum] and develop alongside one another during childhood (McClelland and Cameron, 2019). For example, Koutsandréou et al. (2016) evaluated the effectiveness of a 10-week endurance and coordination training program on working memory performance in 9–10-year-olds. After 10 weeks, the group that had completed coordinative tasks (e.g., balancing and bilateral exercises) benefited most from the training, and significantly outperformed the control group in a complex memory task that focused on the central executive component of working memory.

Despite close links between motor and cognitive domains in early childhood (McClelland and Cameron, 2019; Gordon-Murer et al., 2021; Stuhr et al., 2020), preschool aged children (i.e., 5- to 6-year-old children) are still underrepresented in the existing research and literature. This is unfortunate given that the preschool years lay the foundation of EF development (Demetriou et al., 2020; Vandenbroucke et al., 2017), with inhibition and working memory emerging as distinct but interrelated factors during this time period (Miller et al., 2012; Monette et al., 2015). Therefore, the aim of the present study was to examine the effectiveness of a 4-week manual dexterity training program to improve working memory performance, cognitive functioning, and numeracy skills in 5- to 6-year-old preschool children. Based on existing literature highlighting the strong interrelations between manual dexterity and working memory in preschool children (McClelland and Cameron, 2019; Michel et al., 2011; Stöckel and Hughes, 2016; Stuhr et al., 2020), it is hypothesized that the manual dexterity training program will lead to greater working memory performance, compared to children in the control group.

A secondary exploratory aim of the present study is to examine whether a 4-week manual dexterity training program leads to improvements in other related abilities, namely inhibition, cognitive flexibility, and numeracy skills. At present, the literature has reported mixed findings (Lakes and Hoyt, 2004; Ludyga et al., 2019). Among intervention studies, there are few that included preschool-age children. For example, Lakes and Hoyt (2004) reported small to moderate effects on inhibition performance after a Tae-Kwon-Do training program that adapted the difficulty level of the sports routine in 5–11-year-old children. In contrast, Ludyga et al. (2019) did not reveal any effect of coordinative training program or physical endurance training on inhibition performance in 9- to 10-year-old children. However, it should be noted that Ludyga et al. (2019) did not adjust the difficulty level over time, which may account for the null findings.

Recent empirical reviews emphasize that training interventions that directly address specific EFs do not benefit non-trained EF and academic domains (Diamond and Ling, 2016; Redick et al., 2015; Tomporowski et al., 2015). However, what is not known is whether motor skill interventions that incorporate components that directly train and challenge EFs (i.e., the so-called direct route) that also create joy, pride, and a social sense of belonging (i.e., the so-called indirect route) (Diamond, 2014) can lead to positive benefits to non-trained cognitive and academic skills. According to Diamond (2014), such an approach could reduce stress, which is known to be one of the key components for impaired EFs. Consequently, a sensorimotor program that also supports social, mental, and personal skill development should have a stronger impact on overall cognitive and EF development.

Standard statistical methodologies that compare time-by-group changes [e.g., repeated measures analysis of variance (RM ANOVA)] provide information on whether intervention groups, as a whole, exhibit statistically significant improvements when compared to a control group. While average-based change approaches provide valuable information regarding the efficacy of a treatment, they do not identify individuals that exhibit meaningful change. This is unfortunate, given that determining for whom meaningful changes in performance have occurred is a key goal in cognitive and sensorimotor intervention studies. A common approach to measuring individual-level clinically significant responsiveness to an intervention is via the Jacobson-Truax reliable change index (RCI, Jacobson et al., 1984; Jacobson and Truax, 1991). This methodology first determines whether the observed pre- to post-test change is statistically reliable (rather than an artifact of measurement error), with a cut-off point marking the transition between a patient/functional population and a non-patient/dysfunctional population (i.e., clinical significance). Subsequently, individuals are classified into clinical outcome categories: “deteriorated,” “unchanged,” and “improved.” As such, the Jacobson-Truax RCI examines the statistical, *as well as* the clinical, significance of an intervention, while accounting for the reliability of measures used to capture the change. The use of the Jacobson-Truax RCI method has been widely used in psychotherapy research (Ronk et al., 2016), and has also been applied to cognitive interventions for neuromotor impairments [e.g., multiple sclerosis (Hancock et al., 2015), sports-related mild traumatic brain injury (Echemendia et al., 2012), as well as developing children (Moore et al., 2018; Vardanian et al., 2020)].

Given these benefits, the present study examined whether clinically meaningful changes can be detected using the RCI presented by Jacobson and Truax (1991). Adopting statistical approaches that evaluate individual-based clinical change has benefits for both the research and education communities. First, it provides the academic community with an entry point for exploring sources of intervention-based heterogeneity. Second, individual-based change measures can provide educators with the means by which to identify students who require remediation, as well as identify students who are excelling and require more challenge.

## 2 Methods

### 2.1 Participants

Forty-five 5- to 6-year-old children from two different local preschool facilities participated in this study (age range = 62–79 months, mean age = 69.28 ± 4.99 months; 25 boys). Children were quasi-randomly assigned to either an intervention (*n* = 20, mean age = 69 months, 10 boys) or a control group (*n* = 25, mean age = 70.40, 10 boys) according to their kindergarten affiliation. All participants had normal or corrected to normal vision, and normal hearing. Participants were excluded if they had any known neurological or physical disorders that could impair their ability to perform activities of daily living, had an Individualized Education Plan (IEP), or were unable to speak and understand German. The experiment was approved by the local school authorities and the institutional review board, and conformed to the declaration of Helsinki.

### 2.2 Design

This study used a 4-week parallel-groups design. Potential participants underwent in-person screening for visual acuity, language comprehension, and learning disabilities prior to enrollment in the study. Children were quasi-randomly assigned to either an intervention or a control group according to their kindergarten affiliation. Treatment allocation was concealed to maintain blinding. To ensure impartiality, the individual responsible for group assignment had no involvement in data collection, with only the persons administering treatment (i.e., evaluators) aware of group assignment.

After meeting the eligibility criteria, participants underwent baseline evaluation (pre-test) of cognitive and motor performance, as well as numeric ability. The assessment battery spanned 2 days, with sessions lasting 30–40 min each, and tests were administered in a randomized order for all participants. Within 1 week of completing the treatment protocol, participants completed the same evaluation procedure again (post-test). All pre- and post-intervention assessments were conducted in a quiet environment, consistently scheduled between 10 a.m. and noon, and overseen by an examiner blinded to the participants' group assignments. Children were guided through the test stations using a treasure map to motivate them through the testing procedure.

All evaluators completed a comprehensive training program prior to receiving approval to conduct the cognitive and motor assessments. The training protocol follows standard procedures in our laboratories and involves individualized training sessions conducted by an experienced experimenter, and consists of initial presentation of each task individually, guided practice with mock participants, addressing questions and concerns, troubleshooting, and evaluation.

### 2.3 Treatment protocol

Over the 4 weeks following baseline testing, children in the intervention group (IG) participated in manual dexterity training, while children in the control group (CG) were read to. Both groups received 12 treatment sessions (three per week), each lasting 30–40 min.

#### 2.3.1 Manual dexterity intervention

The manual dexterity training program was designed to fit within the practical constraints of preschool settings, considering time and resource limitations. The intervention was structured to maximize engagement while being feasible for teachers to implement within the classroom routine. Children were trained in small groups of four to foster peer interaction (cf. Diamond, 2012; Diamond and Ling, 2016) while also promoting individual learning. The tasks were brief and varied, allowing integration into the daily schedule without requiring extensive preparation or resources. Additionally, structured feedback and teacher reinforcement ensured that children received guidance throughout the program, fostering positive learning experiences and skill development. The program focused on fine motor tasks that were visually controlled and integrated social interaction and peer learning. This design aligns with research suggesting that interventions incorporating social, cognitive, and interactive components enhance executive function outcomes in preschool-aged children (Diamond, 2012; Diamond and Ling, 2016).

The intervention involved structured age-appropriate “Pirate Training” sessions, where the groups of children engaged in five different tasks based on validated measures of manual dexterity [i.e., the Purdue Pegboard task (Tiffin and Asher, 1948), the Bruininks-Oseretsky Test of Motor Proficiency (BOT, Blank et al., 2014)]. At three out of the five stations, tasks were kept consistent across training sessions, maintaining the same context and tasks (constant stations). However, to introduce variety and prevent monotony, one aspect of the tasks at two stations was alternated between sessions, offering some variation in the intervention and mitigating boredom associated with repetitive activities (changing stations). The complexity of tasks for all children in the IG was increased from weeks 1 to 3. This involved increasing accuracy demands, for example by using smaller beads during threading tasks or more difficult shapes in cutting tasks. In week 4, all tasks from the previous 3 weeks were repeated starting with those from week 1. The tasks at each of the five stations were performed three times for 1 min each, with a break of 1 min between stations.

Children in the IG performed the tasks individually and were awarded colored LEGO^®^ blocks after completing each station to encourage motivation. Specifically, three Legos were given if performance was quantitatively better (or if they qualitatively performed with more effort), than during their previous attempt at the task, two Lego blocks if performance remained constant, and one Lego block if performance decreased regardless of effort. The intervention supervisor gathered the building blocks contributed by all children in the group and constructed a single tower. A group discussion followed each session, where progress was reviewed, feedback was provided, and praise was given, reinforcing both individual and group achievements. During this session, the supervisor offered praise, encouragement, and constructive feedback to motivate the children further. In this way, the children (individually and as a group) were always able to compare their tower with the one from previous days, enabling a sense of collective achievement in building the tower together while also recognizing their own contributions to the group. At the end of the 4-week training program, children were officially welcomed into the “Pirate Team,” which included the awarding of pirate names and handing out of eye patches.

#### 2.3.2 Control activity

For the control group, children were exposed to passive listening activities, such as stories and fairy tales, read by their kindergarten teachers. This control condition was intentionally chosen to minimize motor and cognitive demands, ensuring structured participation through passive story listening, a method commonly used in developmental research to engage children without requiring fine motor manipulation or complex cognitive processes (cf. Diamond and Ling, 2016). While this control condition did not involve fine motor skills, it provided a consistent, low-interaction alternative that was easily implemented within the preschool environment with minimal teacher involvement, beyond routine classroom management.

### 2.4 Outcome measures

Pre- and post-test EF and motor performance were assessed using tasks with demonstrated reliability and validity in young children. Manual dexterity was evaluated using the Purdue Pegboard Test and the manual dexterity subtest of the Bruininks-Oseretsky Test of Motor Proficiency (BOT-2). Working memory tests included a sequencing test, the Corsi-Block-Tapping test, and the verbal short-term memory Language Development Test for 3- to 5-year-olds (a subtest of the SETK 3-5). Additionally, the Flanker task was used to assess selective attention, the Hearts and Flowers test was used to assess response inhibition and cognitive flexibility, a simple reaction time test was used to measure processing speed, and the preschool version of the TEDI-MATH was used to assess precursor mathematical knowledge.

#### 2.4.1 Motor performance

The Purdue Pegboard Test (#32020, Lafayette Instruments, IN, USA, Tiffin and Asher, 1948) and the manual dexterity subtest of the BOT-2 (BOT-2, Blank et al., 2014; Bruininks, 2005) were used to assess manual dexterity. For the Purdue Pegboard Test, participants had to fill a pegboard consisting of two vertical rows of 25 holes with pins as quickly as possible in the given time (30 s for unimanual tasks, 60 s for bimanual task). Participants first used the dominant hand to place as many pins as possible in the holes, then the non-dominant hand, and finally with both hands simultaneously to place two pins in the parallel rows at the same time. Each condition started with a practice trial where children inserted five pins into the holes. The number of inserted pins (or pairs of pins) was averaged across the three trials, after which the sum of the three mean values was calculated to derive the pegboard manual dexterity score. The manual dexterity subtest of the BOT-2 consists of five tasks: dotting circles, transporting coins, inserting pens, sorting cards, and threading dice. For each of the tasks, the number of items that were inserted/transported/punctured in 15 s was documented by the experimenter, which were then summed to derive a BOT manual dexterity total score.

#### 2.4.2 Cognitive performance

##### 2.4.2.1 Visuospatial short term working memory

The Corsi Block-Tapping Test (CBT; Corsi, 1972; Kessels et al., 2000) was used to measure visuospatial (short-term) working memory. At the start of each trial, a static spatial array of blue-colored blocks was displayed on the screen against a black background. The blocks transitioned in color from blue to yellow in a predetermined sequence. Subsequently, participants were required to replicate the sequence by tapping on the blocks in the exact order they were illuminated with a touch pen. The test started with a sequence length of two blocks and increased by one after successful reproduction of at least one of two sequences per sequence length (up to a maximum of nine blocks). If both trials of a sequence length could not be reproduced correctly, the test was discontinued. The total score (up to a maximum of 144), defined as the block span (i.e., sequence length at which the test was terminated) multiplied by the number of correctly repeated sequences until the test was discontinued (i.e., number of correct trials), was used as dependent variable for visuospatial short term working memory.

##### 2.4.2.2 Verbal short term working memory

A subtest from a German language development test for 3- to 5-year-old children (Sprachentwicklungstest für 3- bis 5-jährige Kinder*;* SETK 3-5; Grimm et al., 2010) was used to assess verbal short-term memory (i.e., the phonological loop). The experimenter presented the children with short age-appropriate words at intervals of ~1 s, which had to be subsequently reproduced from memory. The test started with two words and the number of words increased by one word (up to a maximum of seven words) when at least one of two attempts was successful. The SETK memory span represents the span of words participants can reliably reproduce, and was computed by dividing the number of all correctly reproduced attempts by two and adding two (as children started a sequence length of two). The dependent variable of verbal short term working memory thus ranged between zero and eight (i.e., maximum span if participants are correct on each attempt of the two to seven item word lists).

##### 2.4.2.3 Central executive working memory

The central executive of working memory was assessed using a modified object sequencing task (based on the letter-number-sequencing test, Wechsler Intelligence Scale for Children - Fifth Edition; WISC-V; Petermann, 2017), in which different items (either food or animals) were presented to the children on a computer screen at 2-s intervals and accompanied by verbal naming of the respective items. Participants were asked to memorize the presented items and repeat them in order (from small to large) after all items of one sequence had been presented. The test started with two items. After each successful sequence, the number of items increased by one (up to a maximum of seven items). In a second condition, items of two different categories (food and animals) were presented in each sequence of items. Again, participants were asked to sort the items by size, but by categorizing all the items of one category first before continuing with the items of the other category. For each trial that was successfully completed in the first attempt participants were awarded two points. For the correct answer in the second attempt participants were awarded one point. The sum of the points achieved from both conditions (maximum total score was 28 points) was used as a dependent variable for the central executive of working memory.

##### 2.4.2.4 Selective attention

A Flanker task was used to measure selective attention (McDermott et al., 2007; Zaitchik et al., 2014). In each trial, participants placed their hands on a custom-built handlebar located in front of a touch screen monitor (Philips 231C5TJKFU/00). After a fixation cross was presented for 500 ms, five blue fish were presented in a row in the middle of the screen and the participant was asked to react as quickly as possible to the direction the middle fish was facing by pressing the right or left arrow key on the touch screen. Participants performed 51 trials (three blocks of 17 trials each) comprised of four conditions (neutral, no distracting fish, congruent, incongruent) in random order. In the congruent and incongruent conditions, the middle fish was surrounded by (distracting) fish facing in the same or opposite direction as the middle fish, respectively. In the control conditions, the flanking fish were replaced by dashes (neutral) or spaces (no distracting fish). The dependent variable was the accuracy measure across all conditions. Trials with responses faster than 250 ms or two standard deviations above the individual mean were not included in the data analysis.

##### 2.4.2.5 Response inhibition

Self-regulation of inhibitory control was assessed using the Hearts and Flowers Task (Diamond et al., 2007; Wright and Diamond, 2014). Each trial started with a fixation cross in the middle of the screen. Stimuli (a heart or a flower) appeared for 1,500 ms with an interstimulus interval of 500 ms. In the first block (congruent condition) that featured a total of 12 trials, hearts were presented on either the right or left side of the screen. The children were asked to press the button on the same side as the stimulus as quickly as possible. In the second block (incongruent condition, total 12 trials) flowers appeared instead of hearts. Participants were asked to react as quickly as possible by pressing the key on the opposite side of the stimulus. The difference in accuracy (percent correct responses) between the incongruent (flowers) and the congruent conditions (hearts) was used as the dependent variable. Trials with responses faster than 250 ms or two standard deviations above the individual mean were not included in the data analysis.

##### 2.4.2.6 Cognitive flexibility

The third block (mixed condition, total 49 trials) of the Hearts and Flowers task (Diamond et al., 2007; Wright and Diamond, 2014) was used to assess cognitive flexibility. Participants were shown hearts and flowers in a randomized order and had to press the same side of the touchscreen when a heart appeared, and the opposite side of the touchscreen when a flower appeared. As such, the task requires constant switching between two rules and therefore includes aspects of cognitive flexibility (Diamond, 2013). The difference in accuracy (in percent) between hearts (same side response) and flowers trials (opposite side response) was used as measure of cognitive flexibility.

##### 2.4.2.7 Processing speed

A Simple Reaction Time task was used as a measure of processing speed (Kiselev et al., 2009). At the start of each trial, a fixation cross was displayed for 500 ms, and after a random intertrial interval (500–2500 ms) the stimulus (i.e., a red dinosaur) was presented in the middle of the touchscreen monitor. Participants were required to respond as quickly as possible to the appearance of the dinosaur by clicking the left mouse button. A total of 32 stimuli were presented. The average reaction time (across the 32 trials) was used as dependent variable.

##### 2.4.2.8 Precursor mathematical skills

The German version of the preschool TEDI-MATH test (Kaufmann et al., 2009; Van Nieuwenhoven et al., 2001) was used to evaluate early numerical skills. The TEDI-MATH includes the following five subtests: counting principles, counting, visual and auditory number recognition, and calculating with objects. Children received one point for each correct answer in all subtests (maximum total score was 53 points), with the grand sum of points serving as the outcome variable.

### 2.5 Statistical analysis

The clinical significance of change in outcome variables was estimated by the Jacobson–Truax RCI (Jacobson and Truax, 1991). The RCI determines whether an individual's change in score between the pre-test (*x*_1_) and post-test (*x*_2_) is statistically reliable and not due to random measurement error. The RCI is calculated as:


$$\begin{array}{l} RCI=\;\frac{x_{2}-x_{1}}{SE_{diff}} \end{array}$$

where *x*_2_ – *x*_1_ represents an individual's change in score from pre-test (*x*_1_) to post-test (*x*_2_), and *SE*_*diff*_ is the standard error (SE) of the difference between pre- and post-test scores. The standard error of the difference (*SE*_*diff*_), accounts for the reliability of the measurement instrument and is calculated as:


$$\begin{array}{l} SE_{diff}=\;\sqrt{{2\left(SE\right)}^{2}} \end{array}$$

where *SE* is the standard error of measurement for the pre-test, and is calculated using the formula:


$$\begin{array}{l} SE=\;s_{1}\sqrt{1-r_{xx}} \end{array}$$

In this equation, s_1_ represents the standard deviation of the pre-test scores, and *r*_*xx*_, the internal reliability of the measurement calculated using Cronbach's alpha (Lambert and Bailey, 2012). The use of Cronbach's alpha accounts for the internal consistency of the measurement tool, which ensures that the reliability of the scale is appropriately factored into the analysis. By focusing on the pre-test standard deviation, we capture the baseline variability, which is typically more stable and provides a meaningful reference point for measuring change. Incorporating *r*_*xx*_ adjusts for measurement error, thus ensuring that the observed change is not attributable to random fluctuations but reflects true and meaningful difference.

A change is considered statistically reliable if the RCI is > 1.96 (for 95% confidence), indicating the change is real and not likely due to some random measurement error. In this study, participants were classified into different categories: reliable negative change (RCI < −1.96), absence of change (RCI −1.96 to 1.96), and reliable positive change (RCI > 1.96). Unlike group-level statistical tests that compare means across multiple conditions, the RCI evaluates change at the individual level. Because each participant's score is assessed independently, there is no accumulation of Type I error across multiple comparisons, eliminating the need for correction methods typically required in group-level analyses. To test for differences between groups in terms of reliable change, Chi-squared tests were performed to compare the proportions of participants showing reliable positive change, reliable negative change, absence of change. The chi-squared test is appropriate for comparing categorical outcomes, such as the different classifications of change based on the RCI.

In addition, Chi-squared tests were conducted to compare the percentage of participants exhibiting improvement across all measures (range 0–10), manual dexterity (range 0–2), and working memory (range 0–3) between study groups. To further understand the impact of the manual dexterity training program, Spearman rank-order correlation coefficients were calculated between the overall number of tasks with reliable improvement and the number of manual dexterity tasks, separately for each group. This analysis aimed to explore the relationship between the breadth of improvement and the focus on manual dexterity, particularly assessing whether these correlations were distinct for the control group.

## 3 Results

### 3.1 Descriptive statistics

There were no significant differences between groups with respect to age, gender, body mass index (BMI), or electronic device use. On average, participants in the control group had spent more time in day-care (*p* = 0.005) but spent less time per week engaging in physical activity (*p* = 0.003), compared to individuals in the intervention group (Table 1).

**Table 1:** Demographic subject characteristics of children in the control (CG) and intervention groups (IG).

	**Control group (*n* = 25)**	**Intervention group (*n* = 20)**	***p***	***F***	**η^2^**
Males, *n* (%)	10 (40)	10 (50)	0.502	–	–
Age, months	70.40 (5.33)	69.00 (4.22)	0.343	0.918	0.021
BMI, kg/m^2^	14.93 (1.85)	14.73 (1.91)	0.747	0.105	0.003
Time in daycare, months	56.92 (5.60)	48.70 (12.46)	0.005	8.716	0.169
Physical activity, min/week	88.20 (72.76)	202.25 (159.39)	0.003	10.192	0.192
Electronic device use, min/day	38.42 (20.08)	41.60 (31.03)	0.680	0.173	0.004

Table 2 presents the descriptive statistics for the control and intervention groups at pre- and post-test across the 10 dependent variables, with a focus on individual differences. The results from the repeated-measures ANOVA (Group × Time), with Bonferroni correction, are provided in Supplementary material and offer further insights into the Group × Time interactions, highlighting trends in how individual differences may shape outcomes. Generally, the group-level analysis indicated that the IG demonstrated greater improvements in manual dexterity compared to the CG, particularly at post-test. For example, significant main effects of both Group and Time were observed for Purdue Pegboard and BOT manual dexterity tasks (both *p*'s < 0.001), with a notable Group × Time interaction effect for BOT manual dexterity (*p* = 0.027, η^2^p = 0.06) and a near significant interaction for Purdue Pegboard (*p* = 0.070, η^2^p = 0.04), suggesting that the intervention led to greater gains in fine motor skills at the group level. For executive functioning measures, mixed results were observed in the group-level analysis. Significant main effects of Time were found for verbal short-term memory (*p* = 0.004, η^2^p = 0.09), selective attention (*p* < 0.001, η^2^p = 0.26), processing speed (*p* = 0.048, η^2^p = 0.04), and precursor mathematical ability (*p* = 0.009, η^2^p = 0.08), indicating overall improvements across both groups. However, no significant Group × Time interactions were found for working memory or cognitive flexibility, suggesting that these improvements were not specific to the intervention at the group level. Individual differences in clinically significant improvements will be examined in the subsequent sections using RCI analysis.

**Table 2:** Descriptive statistics of pre-test and post-test scores of children in the control (CG) and intervention groups (IG).

	**Control group**		**Intervention group**	
	**Pre-test**	**Post-test**	**Pre-test**	**Post-test**
MD_PP_	27.49 (3.47)	28.88 (4.42)	28.72 (3.30)	32.97 (3.25)
MD_BOT_	17.48 (3.55)	18.80 (3.37)	19.80 (2.97)	24.55 (4.38)
WM_LS_	10.12 (2.68)	10.60 (2.80)	9.20 (2.17)	10.95 (3.32)
WM_CBT_	18.12 (12.01)	18.48 (9.04)	16.15 (6.48)	21.85 (11.38)
WM_SETK_	4.70 (0.68)	4.98 (0.57)	4.75 (0.60)	5.28 (0.68)
SA_acc_	0.76 (0.15)	0.87 (0.10)	0.71 (0.12)	0.88 (0.07)
RI_acc − diff_	−0.04 (0.22)	−0.09 (0.10)	−0.08 (0.15)	−0.05 (0.13)
CF_acc − diff_	−0.04 (0.17)	−0.02 (0.11)	−0.01 (0.17)	0.01 (0.09)
SRT	485.07 (102.49)	455.17 (79.57)	493.11 (85.65)	451.49 (58.22)
TEDI	38.16 (6.04)	41.72 (4.20)	37.21 (7.62)	39.95 (5.57)

### 3.2 Jacobson-Truax reliable change index

The RCI specifies the amount of change individuals must show on each outcome measure for that change to be reliable (i.e., larger than would be expected due to measurement error alone). The RCI was calculated for each participant of the control group and the intervention group from pre-test to post-test, separately for each outcome measure. Table 3 presents Jacobson-Truax RCI values between baseline and post-intervention for all measures of interest.

**Table 3:** Clinically significant improvement measured by Jacobson-Truax reliable change index scores between baseline and post-intervention for all measures of interest.

	**Reliable negative change**	**No reliable change**	**Reliable positive change**	**Chi-square**	***P*-value**	**Clinical cutoff**
Manual dexterity PPB				26.133	* < 0.001*	34.9
Control	0 (0%)	20 (80%)	5 (20%)			
Intervention	0 (0%)	9 (45%)	11 (55%)			
Manual dexterity BOT				26.721	* < 0.001*	25.4
Control	0 (0%)	22 (88%)	3 (12%)			
Intervention	0 (0%)	11 (55%)	9 (45%)			
Central executive WM				6.365	*0.041*	14.7
Control	2 (8%)	19 (76%)	4 (16%)			
Intervention	2 (10%)	12 (60%)	6 (30%)			
Visuospatial WM				19.048	* < 0.001*	37.2
Control	2 (8%)	22 (88%)	1 (4%)			
Intervention	0 (0%)	16 (80%)	4 (20%)			
Verbal short term WM						6.0
Control	0 (0%)	20 (80%)	5 (20%)	19.78	* < 0.001*	
Intervention	0 (0%)	10 (50%)	10 (50%)			
Selective attention						1.0
Control	0 (0%)	19 (76%)	6 (24%)	9.758	*0.008*	
Intervention	0 (0%)	11 (55%)	9 (45%)			
Response inhibition						0.3
Control	5 (20%)	17 (68%)	3 (12%)	1.077	0.584	
Intervention	3 (15%)	14 (70%)	3 (15%)			
Cognitive flexibility				5.343	0.069	0.3
Control	3 (12%)	18 (72%)	4 (16%)			
Intervention	1 (5%)	17 (85%)	2 (10%)			
Processing speed				0.102	0.950	304
Control	0 (0%)	21 (80%)	4 (16%)			
Intervention	0 (0%)	17 (85%)	3 (15%)			
Precursor mathematical skill				2.407	0.300	51.0
Control	0 (0%)	23 (92%)	2 (8%)			
Intervention	0 (0%)	17 (85%)	3 (15%)			

Statistically significant *p* values are shown in italics.

#### 3.2.1 Manual dexterity

A clinically significant cut-off of 34.9 was established for the Purdue Pegboard manual dexterity measure. Eleven out of 20 (55%) participants in the intervention group had an RCI > 1.96, suggesting an improvement in manual dexterity scores. In contrast, only five out of 25 (20%) participants in the control group presented a reliable positive change in manual dexterity scores. Chi-square analysis indicated that there was a significant difference between the proportion of participants in the control and intervention groups who fell into each category [$\chi_{\left(2\right)}^{2}$ = 26.133, *p* < 0.001], with the intervention group having significantly more children with improved manual dexterity during post-test compared to the control group.

A clinically significant cut-off of 25.4 was established for the BOT manual dexterity measure. A total of 11 participants in the intervention group (55%) had no change in performance, and 9 (45%) showed change that surpassed the threshold to be considered reliable change. By contrast, 22 (88%) participants in the control group had no change in performance, and 3 (12%) presented a reliable positive change. Chi-square analysis indicated that there was a significant difference between the proportion of participants in the control and intervention groups who fell into each category [$\chi_{\left(2\right)}^{2}$ = 26.721, *p* < 0.001], with the intervention group having significantly more children with improved BOT manual dexterity scores during post-test compared to the control group.

#### 3.2.2 Working memory

A clinically significant cut-off of 14.7 was established for the central executive working memory measure. The proportion of children demonstrating reliable improvement in central executive working memory was significantly greater for the intervention (*n* = 6, 30%) than the control group (*n* = 4, 16%), $\chi_{\left(2\right)}^{2}$ = 6.365, *p* = 0.041.

A clinically significant cut-off of 37.2 was established for the visuospatial working memory measure. Reliable and clinically significant change of visuospatial working memory from pre- to post-test were as follows for the control group: 8% (*n* = 2) exhibited negative change, 88% (*n* = 22) had no change, and 4% (*n* = 1) achieved clinically significant improvement. In contrast, 80% (*n* = 16) of participants in the intervention group had no change, and 20% (*n* = 4) achieved clinically significant improvement. This difference in frequencies of outcome was not statistically significant, $\chi_{\left(2\right)}^{2}$ = 19.048, *p* < 0.001.

A clinically significant cut-off of 6.0 was established for the verbal short term memory measure. Reliable and clinically significant change of verbal short-term memory from pre- to post-test were as follows for the control group: 80% (*n* = 20) exhibited no change, and 20% (*n* = 5) achieved clinically significant improvement. In contrast, 50% (*n* = 10) of participants in the intervention group had no change, and 50% (*n* = 10) achieved clinically significant improvement. This difference in frequencies of outcome was statistically significant, $\chi_{\left(2\right)}^{2}$ = 19.780, *p* < 0.001.

#### 3.2.3 Secondary measures

The clinically significant cut-off value of 1.0 was established for the selective attention measure. A total of 11 participants in the intervention group (55%) had no change in performance, and 9 (45%) showed change that surpassed the threshold to be considered reliable change. By contrast, 19 (76%) of participants in the control group had no change in performance, and 6 (24%) presented a reliable positive change. This difference in frequencies of outcome was statistically significant, $\chi_{\left(2\right)}^{2}$ = 9.758, *p* < 0.008.

Response inhibition yielded a cut-off value of 0.3, with 15% of participants in the intervention group demonstrating reliable positive change, 70% exhibiting no change, and 15% exhibiting negative change. In contrast, 12% of participants in the control group exhibited reliable positive change, 68% exhibited no change, and 20% exhibited negative change. This difference in frequencies of outcome was not statistically significant, $\chi_{\left(2\right)}^{2}$ = 1.077, *p* = 0.584.

A clinically significant cut-off of 0.3 was established for the cognitive flexibility measure. Reliable and clinically significant change of cognitive flexibility from pre- to post-test were as follows for the control group: 12% (*n* = 3) exhibited negative change, 72% (*n* = 18) had no change, and 16% (*n* = 4) achieved clinically significant improvement. In contrast, 5% (*n* = 1) of participants in the intervention group exhibited negative change, 85% (*n* = 17) had no change, and 10% (*n* = 2) achieved clinically significant improvement. This difference in frequencies of outcome just failed to reach statistical significance, $\chi_{\left(2\right)}^{2}$ = 5.343, *p* = 0.069.

A clinically significant cut-off of 304 was established for processing speed. A total of 17 participants in the intervention group (85%) had no change in performance, and 3 (15%) showed change that surpassed the threshold to be considered reliable change. By contrast, 21 (80%) of participants in the control group had no change in performance, and 4 (16%) presented a reliable positive change. A Chi-Square test revealed that the difference between reliable changes in the two groups was non-significant, $\chi_{\left(2\right)}^{2}$ = 0.102, *p* = 0.950.

A clinically significant cut-off of 51.0 was established for precursor mathematical ability. A total of 17 participants in the intervention group (85%) had no change in performance, and 3 (15%) showed change that surpassed the threshold to be considered reliable change. By contrast, 23 (92%) of participants in the control group had no change in performance, and 2 (8%) presented a reliable positive change. A Chi-Square test revealed that the difference between reliable changes in the two groups was non-significant, $\chi_{\left(2\right)}^{2}$ = 2.407, *p* = 0.300.

### 3.3 Patterns of improvement across tasks

**Figure 1:**
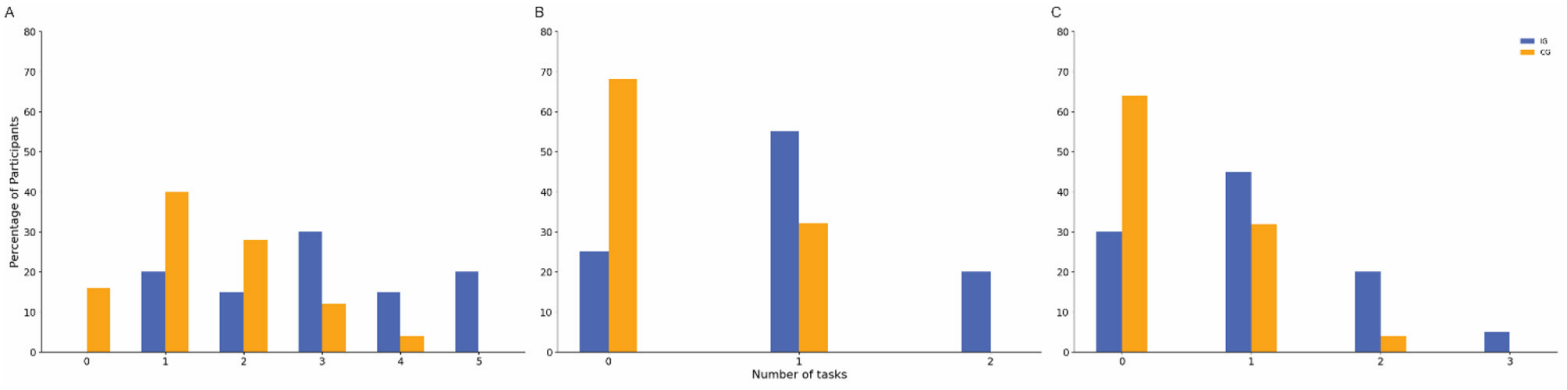
Percentage of children who exhibited statistically and clinically significant improvement in **(A)** all measures, **(B)** manual dexterity tasks, and **(C)** working memory measures. Yellow bars refer to participants in the control group (CG), whereas blue bars refer to participants in the intervention group (IG).

Next, we assessed whether the number of tasks that children who demonstrated statistically and clinically significant improvement differed between the two groups (Figure 1). Overall, 20% of children in the intervention group (*n* = 4) demonstrated improvement in five tasks, 15% (*n* = 3) exhibited improvements in four tasks, 30% (*n* = 6) improved in three tasks, 15% (*n* = 3) improved in two tasks, and 20% (*n* = 4) improved in one task. In contrast, none of the participants in the control group improved in five tasks, 4% improved in four tasks (*n* = 1), 12% (*n* = 3) improved in three tasks, 28% (*n* = 7) improved in two tasks, 40% (*n* = 10) improved in one task, and 16% (*n* = 4) did not improve in any measured task. A Chi-Square test revealed that the difference between reliable changes in the two groups was significant, $\chi_{\left(5\right)}^{2}$ = 60.680, *p* < 0.001.

With respect to manual dexterity measures (Figure 1B), 20% of participants in the intervention group (*n* = 4) reliably improved in both, 55% (*n* = 11) showed improvement in one manual dexterity task, and 25% (*n* = 5) did not improve in either task. In contrast, none of the participants in the control group demonstrated improvement in both tasks, 32% (*n* = 8) showed improvement in one task, and 68% (*n* = 17) did not improve in either task. This difference in frequencies of outcome was statistically significant, $\chi_{\left(2\right)}^{2}$ = 51.890, *p* < 0.001.

With respect to working memory measures, 5% of participants in the intervention group (*n* = 1) reliably improved in all three tasks, 20% (*n* = 4) showed improvement in two tasks, 45% (*n* = 9) improved in one task, and 30% (*n* = 6) did not improve in either task. None of the participants in the control group improved in all three tasks, 4% (*n* = 1) improved in two tasks, 32% (*n* = 8) improved in one task, and 64% (*n* = 16) did not improve in any of the three tested working memory tasks. A Chi-Square test revealed that the difference in frequencies was significant, $\chi_{\left(3\right)}^{2}$ = 27.822, *p* < 0.05.

Correlation analysis indicated a weak positive relationship between the number of tasks with reliable improvement and the number of manual dexterity tasks with reliable improvement (ρ = 0.393, *p* = 0.049) and a moderate positive relationship between the number of working memory tasks with reliable improvement (ρ = 0.472, *p* = 0.017) for the control group. In contrast, for the intervention group, a moderate positive correlation between the number of tasks with reliable improvement and the number of manual dexterity tasks with reliable improvement (ρ = 0.576, *p* = 0.008) was observed. No other correlations reached significance.

## 4 Discussion

The first aim of the present study was to examine the statistical and clinical effectiveness of a 4-week manual dexterity training program enriched with social aspects (cf. Diamond, 2012; Diamond and Ling, 2016) to improve manual dexterity abilities and working memory in 5- to 6-year-old preschool children. Furthermore, by including different working memory components (central executive, visuospatial working memory, verbal working memory), we were able to determine whether these improvements were isolated to a single working memory task or if they affected a variety of working memory components.

Not unexpectedly, a greater percentage of children in the intervention group demonstrated reliable improvement in the Purdue Pegboard (control group = 20%, intervention group = 55%) and BOT (control group = 12%, intervention group = 45%) manual dexterity measures. Congruent with our hypothesis, prior observational studies conducted with older children, and prior motor intervention programs (Hsieh et al., 2017; Koutsandréou et al., 2016; van der Niet et al., 2016; Alesi et al., 2016), a greater proportion of children who received the manual dexterity intervention exhibited statistically significant improvements in working memory, compared to children in the control group. In addition, a greater percentage of children in the intervention group demonstrated improvements in both manual dexterity tasks and all working memory tasks, when compared to the control group.

Considering the separate working memory components tested in the present study, a greater percentage of children in the intervention group exhibited a reliable improvement in verbal short term memory scores (50%), compared to central executive (30%), and visuospatial short-term memory (20%). These results are consistent with previous studies investigating the effects of different motor training programs on either a broad range of EFs (Alesi et al., 2016; van der Niet et al., 2016) or working memory performance in particular (Hsieh et al., 2017; Koutsandréou et al., 2016) in early childhood. These studies have reported improvements in working memory performance in the central executive (Koutsandréou et al., 2016; van der Niet et al., 2016) and visuospatial working memory (Hsieh et al., 2017; Alesi et al., 2016). However, contrary to the findings of Alesi et al. (2016), most of the participants in our study showed benefits in verbal short-term memory following the manual dexterity training. Thus, our data indicates that all components of working memory were engaged in some way during our manual dexterity training program, therefore improvements were not restricted to a specific component of working memory. Indeed, it is thought that visually controlled fine motor tasks (e.g., handwriting, object manipulation) are one of the key factors of early child development that contribute to the development of neuronal adaptation, which in turn is used by children to successfully perform cognitive tasks (Adolph, 2005). This notion is supported by MacDonald et al. (2016), who demonstrated that the visual motor integration abilities of 3- to 5-year-old children in autumn/fall were associated with the EFs determined in spring.

The secondary aim was to identify possible transfer effects to other EFs (inhibition, cognitive flexibility, processing speed) and numeracy skills. In addition to working memory, a greater percentage of children who received the intervention exhibited a significantly reliable improvement in selective attention (45%), compared to their peers who passively listened to stories and fairy tales read to them by their preschool teacher (24%). This finding is not unexpected from an applied standpoint, considering that activities involving fine motor precision (e.g., cutting or threading beads, coordinating finger and hand movements in a specific sequence) inherently engage attentional processes. These tasks necessitate both selective attention (i.e., discriminating relevant from irrelevant stimuli) and sustained attention (i.e., maintaining focus over extended periods) to achieve successful outcomes. Ikkai and Curtis (2011) assert that sustained neural activity, as engaged in manual dexterity training, extends beyond working memory performance to encompass various cognitive domains, including attention, spatial memory, and aspects of planning, all processed within overlapping brain regions.

While prior research has established an association between selective attention and manual dexterity (Diamond, 2013), it remains plausible that the enhancement in selective attention may stem from improved working memory performance. This is because working memory performance and selective attention are intricately linked and not easily separable in preschool-aged children (Gazzaley and Nobre, 2012; Ikkai and Curtis, 2011; Reynolds and Romano, 2016). However, previous studies have indicated that working memory training alone does not directly enhance attentional resources (cf. Diamond and Ling, 2016). Hence, we postulate that the manual dexterity training employed in this study directly fosters improvements in attentional resources, distinct from any gains in enhanced working memory performance. That said, it's worth noting that manual dexterity training may also lead to enhancements in manual dexterity, working memory, and attentional resources simultaneously, as these skills appear to be foundational for success in such activities. Future research endeavors could delve deeper to disentangle the specific mechanisms underlying the observed enhancements in attentional resources resulting from manual dexterity training, thus shedding more light on the intricate interplay between manual dexterity, working memory, and attentional processes in preschool-aged children.

Despite the reliable improvement in manual dexterity, working memory, and selective attention, the present training program did not lead to improvements in other cognitive functions (i.e., response inhibition, cognitive flexibility, processing speed) or numerical abilities. This finding is consistent with the expanding body of research indicating that EF training tends to be specific to the trained domain, often resulting in improvements limited to the practiced tasks and components. Such training typically does not yield transfer effects to untrained EFs or academic outcomes (e.g., Diamond and Ling, 2016; Kassai et al., 2019; Melby-Lervåg et al., 2016; Niebaum and Munakata, 2023). In the context of academic achievements, previous studies have consistently demonstrated that while training can enhance working memory performance, the broader transfer effects to mathematical skills have often been negligible or lacking (Fälth et al., 2015; Henry et al., 2014). After reviewing the current body of research, Redick et al. (2015) and Melby-Lervåg et al. (2016) concluded that working memory training does not lead to improvements in academic contexts such as arithmetic skills. Similar to a lack of far transfer effects of manual dexterity (or working memory) training to academic skills, there is also limited empirical support for far transfer effects to other EFs (Diamond, 2013). For example, a study with 4- to 5-year-olds did not find positive effects of working memory training on inhibition or higher EFs such as problem-solving skills (Thorell et al., 2009). The lack of transfer effects observed in this study reinforces the idea that EF training is specific to the practiced task (or components therein), with only EFs being honed that are directly used or practiced (Best et al., 2009; Niebaum and Munakata, 2023; Stuhr et al., 2020).

However, it's important to acknowledge the potential for delayed far transfer effects that may emerge over a longer timeframe following training (Ericsson, 2008; Holmes et al., 2009; Telford et al., 2012). For example, Holmes et al. (2009) reported in their study that working memory training did not lead to improvements in mathematical skills until 6 months after completion of the training. With respect to sensorimotor interventions, demonstrated effects come from studies with a minimum duration of 2 years (Ericsson, 2008; Telford et al., 2012). Based on this evidence, Diamond and Ling (2016) have posited that the beneficial outcomes of such training regimens require substantial time for integration into both sensorimotor and cognitive systems, often manifesting to a limited extent or not at all immediately post-treatment. This phenomenon is particularly notable when interventions target multiple EFs (Bergman Nutley et al., 2011; Diamond and Ling, 2016). An additional consideration is that our study did not directly assess mathematical skills, but instead focused on precursor skills such as counting principles. EFs, such as working memory, typically come into play when tasks are challenging and intricate. It's therefore reasonable to assume that by the age of 5–6 years, skills like counting become automated, thereby diminishing the direct influence of working memory compared to the demands encountered in later-stage mathematical activities in school. Taken together, our data suggests that a manual dexterity training program that includes aspects that foster social belonging is not restricted to improvements in manual dexterity. This broader impact holds particular significance for preschool-aged children, given the documented link between fine motor skills in preschool and subsequent academic achievement in areas such as reading and mathematics during early elementary school years (i.e., grades 2–3, Dinehart and Manfra, 2013; Manfra et al., 2017). Instead, our results suggest that the manual dexterity training program may additionally enhance (a) working memory performance and (b) selective attention. However, the precise elements of the training program responsible for these improvements remain unclear.

To gain deeper insights into the underlying processes, future research should explore one or all of the following potential explanations: (a) Manual dexterity tasks may inherently challenge EFs such as working memory and attentional resources, given that EFs are refined through engagement in everyday tasks (Best et al., 2009; Niebaum and Munakata, 2023). Consequently, improvements in EFs resulting from manual dexterity training might be task specific. (b) The novel and demanding nature of the manual dexterity tasks in our study likely engaged (general) cognitive control processes, including aspects of working memory and selective attention. Therefore, improvements in EFs may extend to other tasks, with children exhibiting lower EFs benefiting most from practice (Diamond and Ling, 2016). (c) The social dynamics inherent in the group setting of our study may have contributed to improvements in working memory and selective attention. This aligns with the notion of an indirect pathway to EF training (cf. Diamond, 2012). Hence, it is expected that the enhancements in EFs resulting from the intervention would extend to various tasks, with children lacking supportive relationships (e.g., at home or in the community) benefiting the most from such training (Diamond and Ling, 2016). We would argue that all three explanations are complementary and likely interact to provide a more comprehensive understanding of our findings. Specifically, the sensorimotor elements of the intervention, coupled with the progressive increase in task demands and the real-life relevance of the exercises, collectively contributed to the observed improvements in manual dexterity, working memory, and selective attention. This multifaceted approach provided a stimulating environment that engaged the entire cognitive system while targeting specific EFs.

The proposed explanations for the observed improvements in EFs in our study align well with the findings of Niebaum and Munakata (2023), who argue that EFs are best trained through contextualized programs that directly engage individuals in solving real-world tasks. In our case, the manual dexterity (pirate) training involved activities such as cutting, tracing lines, and threading beads, all of which required active use of EFs to accomplish the task demands effectively. Furthermore, the intervention's group setting and the use of a treasure map to guide children through the sessions likely fostered feelings of enjoyment and social belonging (c.f., Diamond, 2012, 2014). These positive experiences may have created feedback loops that enhanced both cognitive and sensorimotor functioning, contributing to the observed enhancements in EFs and manual dexterity. Building on this, Braem and Hommel (2019) posited that the absence of transfer effects, alongside other characteristics, suggests that EFs are context-dependent processes shaped by associative learning mechanisms. This perspective portrays EFs not as innate cognitive faculties but rather as “cognitive gadgets” that evolve through social interaction and cultural learning (Braem and Hommel, 2019; Heyes, 2018). This aligns with Diamond's concept of an indirect pathway to enhancing EFs (Diamond, 2012, 2014) and Niebaum and Munakata's (2023) approach of training EF engagement through reinforcement and real-world relevance to address specific environmental demands. Consequently, actively contemplating strategies to solve a specific (motor) problem, especially within cooperative small groups, holds promise for extending the effectiveness of motor interventions to higher cognitive skills, namely the EFs required to effectively tackle the task at hand. In educational settings like in kindergarten or preschool such a training program can be easily and systematically implemented on a daily (or at least weekly) basis. Small groups of three to four children could be given specific (motor) problems, like building something out of paper, beads, building blocks, Legos or materials from nature, that require children to work together as a group and to use their hands for fine motor tasks (e.g., cutting out, stacking blocks, drawing or forming shapes). The given motor problems could be embedded in stories, weeks designated to specific topics or games relevant to the respective group of children.

## 5 Limitations and future directions

While the current study provides useful information for educators and researchers about the efficacy of manual dexterity interventions, several limitations must be acknowledged. The relatively small sample size limits the generalizability of findings to preschool children in other geographic regions. Additionally, the small sample size prevented us from stratifying children based on demographic and home environment factors (e.g., perinatal health, socioeconomic status, family relationships) which could potentially influence sensorimotor and cognitive functioning, as well as learning processes. Future studies should explore the impact of these factors on outcomes to better understand the conditions under which school-based motor and/or cognitive interventions are most effective. Furthermore, as the participants started primary school shortly after the final testing and were distributed across various schools, we were unable to evaluate the long-term benefits of the intervention via follow-up tests. Previous research suggests that some effects of manual dexterity interventions may only become apparent through long-term retention tests due to functional and structural adaptations or reorganization in cognitive and sensorimotor systems. Therefore, future studies should include a 6-month follow-up to examine long-term benefits.

It is important to note that the CG participated in passive story listening, which neither challenged cognitive resources to the same extent nor provided the same level of social interaction and external feedback as the manual dexterity tasks. While passive story listening was selected as a control due to its common use in developmental research as a low-engagement task (cf. Diamond and Ling, 2016), a more appropriate motor-based control condition (e.g., consisting of gross motor activities) should be employed to better isolate the specific aspects of the intervention responsible for the observed improvements. Additionally, future studies could explore additional factors, such as baseline stress levels, feelings of social belonging, or perceptions of self-efficacy, which may influence outcomes. To further enhance our understanding, future research could also benefit from employing multiple measures of each cognitive domain and academic aspect. The task impurity problem in young children (cf. Denckla, 1994) suggests that using only a single measure may not capture the full range of cognitive benefits from the training. By incorporating a variety of measures, researchers can obtain a more comprehensive picture of manual dexterity training benefits.

Despite these limitations, the present study demonstrates the feasibility of conducting manual dexterity training in groups of preschool children, resulting in significant improvements in manual dexterity, working memory, and selective attention measures. While we observed a relatively balanced distribution of boys and girls in each group, the small sample size may limit the generalizability of these findings, particularly regarding potential gender differences in response to the training. Understanding how gender influences the effectiveness of manual dexterity training programs and cognitive outcomes could provide valuable insights for future research. As we look ahead, it will be vital to extend these findings to diverse populations, including typically developing children of various ages, as well as those with cognitive and neurological conditions (e.g., cerebral palsy, developmental coordination disorder).

Moreover, the Jacobson-Truax RCI could provide valuable insights into individual-level treatment effects, allowing for tailored recommendations to support child development. By employing this method, researchers can systematically investigate factors contributing to the motor-cognition interaction on both individual and group levels. Understanding why some children benefit from interventions while others do not, and delineating the differences between responders and non-responders, holds significant implications for optimizing intervention strategies. In the context of the present study, exploring whether children who responded to the treatment differed in personal, social, and/or cognitive skills from non-responders would have provided valuable insights into individual variability in treatment outcomes. Due to the limited sample size of only 20 participants receiving the intervention in this study, the statistical power to detect meaningful differences in baseline abilities is significantly constrained. Consequently, analyses of subgroup differences are likely underpowered, limiting our ability to draw valid conclusions. Future studies with larger sample sizes would be better equipped to effectively explore these differences.

## 6 Conclusion

In summary, our study findings highlight the effectiveness of a four-week manual dexterity training program embedded in a socially enriched group setting, resulting in notable improvements in working memory. While transfer effects were observed for selective attention, improvements did not extend to other cognitive domains such as response inhibition, cognitive flexibility, processing speed, and numerical skills. Importantly, the use of RCI scores provides educators and researchers with a valuable tool for measuring intervention effectiveness and efficacy, allowing for targeted modifications when individuals do not respond as expected to a given treatment. These insights contribute to the ongoing refinement of intervention strategies aimed at optimizing cognitive development in preschool-aged children.

## Data Availability

The raw data supporting the conclusions of this article will be made available by the authors, without undue reservation.
