# Supplementary material for: Effectiveness of a manual dexterity training program to improve executive functioning in preschool children: an individual difference analysis
Source: Front Cognit. 2025 Mar 28;4:1433759. doi: 10.3389/fcogn.2025.1433759 (PMC13281072; doi:10.3389/fcogn.2025.1433759)
Supplement: Supplementary file 1 [file Table_1.docx]

**Supplementary Table 1**. Analysis of variance (ANOVA) table

|  | **Sum of Squares** | **df** | **Mean Squares** | **F** | **p** | **η^2^_p_** |
| --- | --- | --- | --- | --- | --- | --- |
|  |  |  |  |  |  |  |
| **MD_PP_** |  |  |  |  |  |  |
| Group | 156.65 | 1 | 156.64 | 11.56 | *0.001* | 0.12 |
| Time | 159.11 | 1 | 159.11 | 11.74 | *0.001* | 0.12 |
| Group x Time | 45.55 | 1 | 45.55 | 3.36 | 0.070 | 0.04 |
| Error | 1165.37 | 86 | 13.55 |  |  |  |
|  |  |  |  |  |  |  |
| **MD_BOT_** |  |  |  |  |  |  |
| Group | 361.81 | 1 | 361.81 | 28.12 | *< 0.001* | 0.25 |
| Time | 182.04 | 1 | 182.04 | 14.15 | *< 0.001* | 0.14 |
| Group x Time | 65.36 | 1 | 65.36 | 5.08 | *0.027* | 0.06 |
| Error | 1106.39 | 86 |  |  |  |  |
|  |  |  |  |  |  |  |
| **WM_LS_** |  |  |  |  |  |  |
| Group | 1.81 | 1 | 1.81 | 0.24 | 0.629 | 0.00 |
| Time | 24.54 | 1 | 24.54 | 3.20 | 0.077 | 0.04 |
| Group x Time | 8.96 | 1 | 8.96 | 1.17 | 0.282 | 0.01 |
| Error | 658.79 | 86 | 7.66 |  |  |  |
|  |  |  |  |  |  |  |
| **WM_CBT_** |  |  |  |  |  |  |
| Group | 10.89 | 1 | 10.89 | 0.11 | 0.745 | 0.00 |
| Time | 168.10 | 1 | 168.10 | 1.65 | 0.203 | 0.02 |
| Group x Time | 158.42 | 1 | 158.42 | 1.55 | 0.216 | 0.02 |
| Error | 8771.98 | 86 | 102.00 |  |  |  |
|  |  |  |  |  |  |  |
| **WM_SETK_** |  |  |  |  |  |  |
| Group | 0.66 | 1 | 0.66 | 1.66 | 0.201 | 0.02 |
| Time | 3.40 | 1 | 3.40 | 8.55 | *0.004* | 0.09 |
| Group x Time | 0.33 | 1 | 0.33 | 0.84 | 0.363 | 0.01 |
| Error | 34.23 | 86 | 0.40 |  |  |  |
|  |  |  |  |  |  |  |
| **SA_acc_** |  |  |  |  |  |  |
| Group | 0.01 | 1 | 0.01 | 0.40 | 0.530 | 0.00 |
| Time | 0.40 | 1 | 0.40 | 29.65 | *< 0.001* | 0.26 |
| Group x Time | 0.02 | 1 | 0.02 | 1.20 | 0.277 | 0.01 |
| Error | 1.15 | 86 | 0.01 |  |  |  |
|  |  |  |  |  |  |  |
| **RI_acc-diff_** |  |  |  |  |  |  |
| Group | 0.00 | 1 | 0.00 | 0.00 | 0.999 | 0.00 |
| Time | 0.00 | 1 | 0.00 | 0.12 | 0.728 | 0.00 |
| Group x Time | 0.04 | 1 | 0.04 | 1.76 | 0.188 | 0.02 |
| Error | 2.12 | 86 | 0.02 |  |  |  |
|  |  |  |  |  |  |  |
| **CF_acc-diff_** |  |  |  |  |  |  |
| Group | 0.02 | 1 | 0.02 | 0.91 | 0.334 | 0.01 |
| Time | 0.01 | 1 | 0.01 | 0.28 | 0.595 | 0.00 |
| Group x Time | 0.00 | 1 | 0.00 | 0.02 | 0.881 | 0.00 |
| Error | 1.70 | 86 | 0.02 |  |  |  |
|  |  |  |  |  |  |  |
| **SRT** |  |  |  |  |  |  |
| Group | 28.94 | 1 | 28.94 | 0.00 | 0.949 | 0.00 |
| Time | 28326.79 | 1 | 28326.79 | 4.04 | *0.048* | 0.04 |
| Group x Time | 874.36 | 1 | 874.36 | 0.12 | 0.725 | 0.00 |
| Error | 603132.51 | 86 | 7013.17 |  |  |  |
|  |  |  |  |  |  |  |
| **TEDI** |  |  |  |  |  |  |
| Group | 36.98 | 1 | 36.98 | 1.08 | 0.302 | 0.01 |
| Time | 246.68 | 1 | 246.68 | 7.19 | *0.009* | 0.08 |
| Group x Time | 1.74 | 1 | 1.74 | 0.05 | 0.822 | 0.00 |
| Error | 2949.50 | 86 | 34.30 |  |  |  |
|  |  |  |  |  |  |  |
